
JOURNAL INFORMATION
==============================
NLM Title Abbreviation: Tex Med J (Austin)
NLM Journal ID: 21130590R

Texas Medical Journal

ISSN: 0892-8495
Publisher: Biomedical Journal Digitization Project

ARTICLE INFORMATION
==============================
PMCID: PMC9590838
Article version: 1
Subjects: Books and Magazines

Urinalysis

A Guide for the Busy Practitioner

Print publication date: 1898 Dec
Volume: 14
Issue: 6
First page: 345
Last page: 345
License: This work is free of known copyright restrictions. For more information, please see PMC Back Issue Digitization.
License URL: https://creativecommons.org/publicdomain/mark/1.0/

==============================

---

URINALYSIS. A Guide for the Busy Practitioner. By Heinrich Stern, Ph. D., M. D. Published by E. R. Pelton, 129 Fifth Avenue, New York. Pages, 61.

A short concise and practical laboratory guide for the general practitioner. The little book is all that its title page claims for it and will be found very convenient and satisfactory. I. J. J.

---

### Publisher's Notes.

SPEND YOUR VACATION IN THE MOUNTAINS.—But first write the general passenger agent of the Colorado Midland Railroad, Denver, for maps, views, and descriptive matter, so as to know where to go.

---

NEW ORLEANS POLYCLINIC.—Physicians will find the Polyclinic an excellent means for posting themselves upon modern progress in all branches of medicine and surgery. The specialties are fully taught, particularly laboratory work. The 12th Annual Session opens November 24th, 1898. For further information address New Orleans Polyclinic, P. O. Box 797, New Orleans, La.

---

TOURS IN THE ROCKY MOUNTAINS.—The "Senic Line of the World," the Denver & Rio Grande Railroad, offers to tourists in Colorado, Utah and New Mexico the choicest resorts, and to the transcontinental traveler the grandest scenery. Two separate and distinct routes through the Rocky Mountains, all through tickets available by either. The direct line to Cripple Creek, the greatest gold camp on earth. Double daily train service with through Pullman sleepers and tourists' cars between Denver and San Francisco.

The best line to Utah, Idaho, Montana, Oregon and Washington via the "Ogden Gateway."

Write S. K. Hooper, G. P. & T. A., Denver, Colorado, for illustrative descriptive pamphlets.

---

E. N. CAMPBELL, M. D., Good Hope, Ill., says: I have used Aletris Cordial in threatened miscarriages, and find it one of the finest and most efficient preparations that it has been my privilege to prescribe. Aletris Cordial should be used more than it is, although it is largely prescribed, yet like its twin sister, Celerina, it is not prescribed often enough to prove its efficiency. Most all
